# Effect of Exercise Modality on Heart Rate Variability in Adults: A Systematic Review and Network Meta-Analysis

**DOI:** 10.31083/j.rcm2501009

**Published:** 2024-01-09

**Authors:** Faming Yang, Ying Ma, Shuangyan Liang, Yali Shi, Chen Wang

**Affiliations:** ^1^Department of Rehabilitation Sciences, Ningbo College of Health Sciences, 315100 Ningbo, Zhejiang, China; ^2^Department of Rehabilitation Medicine, Linfen Central Hospital, 041000 Linfen, Shanxi, China; ^3^School of Sports Medicine and Rehabilitation, Beijing Sport University, 100084 Beijing, China

**Keywords:** exercise, adults, heart rate variability, network meta-analysis

## Abstract

**Background:**

The purpose of this study was to use a network meta-analysis (NMA) to compare the effects of aerobic training (AT), resistance training (RT), combined training (CBT), and high-intensity interval training (HIIT) on adult heart rate variability (HRV).

**Methods:**

We searched PubMed, the Cochrane Library, Embase, the Web of Science, Wanfang Data, and the China National Knowledge Infrastructure to identify randomized controlled trials on the effects of exercise on HRV in adults. The search was conducted from the outset of these databases to April 2023. Two reviewers independently screened the retrieved articles, extracted raw data from the relevant studies, and assessed the possible risk of bias in the included studies.

**Results:**

The NMA showed that HIIT had the greatest effect on the low-frequency (LF) power/high-frequency (HF) power ratio, standard deviation of normal–normal intervals (SDNN), and root mean square of successive differences between adjacent normal-to-normal intervals (RMSSD) (surface under the cumulative ranking curve (SUCRA) = 99.75%, 98.7%, and 84.9%); CBT had the greatest effect on the LF power (SUCRA = 66.3%); RT had the greatest effect on the HF power (SUCRA = 72.5%).

**Conclusions:**

Our NMA and SUCRA ranking results suggest that in adults, HIIT is the most effective exercise modality in improving the SDNN, RMSSD, and LF/HF power ratio; RT for the HF power; CBT for the LF power. Any NMA conducted in the future must fully explore the effects of different exercise modalities on HRV in adult subgroups of different ages and genders.

**Systematic Review Registration:**

https://www.crd.york.ac.uk/PROSPERO/display_record.php?RecordID=424054, identifier: CRD42023424054.

## 1. Introduction

According to the World Health Organization, approximately 18 million people die of cardiovascular disease (CVD) each year, which accounts for more than 31% of all deaths worldwide, and with a trend toward younger people in recent years [1]. It has been reported that about 30% of the world’s population dies prematurely owing to a lack of physical activity, which accounts for approximately 9% of the total number of people who are physically inactive, thereby making it the largest global public health problem in the 21st century [2]. Physical inactivity is the leading cause of chronic non-communicable diseases and the fourth leading risk factor for increased mortality from non-communicable diseases [3]. Research has found that CVD has become the leading cause of death worldwide as the global population ages [4].

Autonomic function plays an important role in the development of CVD and is a key factor in cardiovascular health and prevention [5]. Among the indicators of autonomic function, heart rate variability (HRV), defined as the difference in adjacent RR intervals between beats in the electrocardiogram over time, responds to sympathetic- and parasympathetic-induced changes in the heart rate. Further, HRV is a non-invasive parameter used to evaluate cardiac autonomic function [6]. Studies have found that the ability of an individual to continuously adapt to changes in the microenvironment and their cardiovascular health is associated with high HRV excitability [7]. Conversely, autonomic dysfunction is associated with higher sympathetic function and lower parasympathetic activity [8]. A relative reduction in HRV is also an independent predictor of CVD risk and all-cause mortality [9, 10]. Studies have confirmed that the important pathophysiological role of HRV abnormalities is reflected in the early stages of essential hypertension, myocardial infarction, and chronic heart failure, leading to an increased risk of death, which is associated with coronary vasoconstriction and increased myocardial oxygen consumption [11, 12, 13, 14]. Thus, the prevention of HRV abnormalities caused by aging or senescence is a challenge that clinicians continue to address.

Regular exercise has been shown to reduce the risk of CVD and premature death and to have a positive impact on cardiovascular health and autonomic function [15, 16, 17, 18]. However, there is some controversy regarding the changes in HRV caused by exercise, which probably relates to the majority of studies illustrating that regular exercise significantly improves autonomic function in people with hypertension, diabetes, and obesity [19, 20, 21], whereas its effect on the general healthy population has remained inconclusive [22, 23]. Recently, several studies have confirmed that aerobic training (AT) improves HRV and reduces cardiovascular risks in healthy adults [24, 25, 26]. Conversely, there is no consensus on the effect of resistance training (RT) on HRV.

A reduced HRV is associated with reduced muscle mass and strength, and resistance exercise is effective in improving abnormal HRV in adults [27, 28, 29]. Some studies have shown that aerobic exercises, when combined with resistance exercises, can improve both endurance and strength at moderate training volumes and durations [30, 31]; however, their effects on autonomic function have not been systematically summarized. In recent years, high-intensity interval training (HIIT) has gained popularity owing to its time-saving and efficient properties and has been shown to improve sympathetic and vagal conditioning [32, 33]. However, it is not yet clear, which of these exercises, is the most appropriate. Therefore, the effects of exercise interventions on HRV in healthy adults must be studied.

Although a large number of randomized controlled trials (RCTs) and systematic reviews have explored the effects of exercise interventions on HRV in adults, indirect comparisons between different exercise interventions have not yet been conducted, and it remains unclear which exercise interventions are optimally effective. A network meta-analysis (NMA) overcomes the limitations of a traditional meta-analysis by allowing optimal ranking of different exercise interventions through performing direct and indirect comparisons. Therefore, the aim of this study was to use an NMA to assess the effects of aerobic, resistance, aerobic combined with resistance, and high-intensity intermittent exercises on HRV in adults, thereby providing an evidence-based basis for exercise to improve cardiac autonomic function and reduce the cardiovascular risks in adults.

## 2. Materials and Methods

### 2.1 Search Strategy

Databases such as PubMed, Embase, the Cochrane Library, the Web of Science, ClinicalTrials.gov, the China National Knowledge Infrastructure, and Wanfang Data were searched using a combination of topic terms and free terms, using a search deadline of April 2023. The search strategy was constructed using the Population, Intervention, Comparison, Outcome, Study Design (PICOS) tool. The English search terms used included the following: sports, training, exercise, heart rate variability, HRV, cardiac autonomic control, autonomic function, parasympathetic activity, parasympathetic nervous system, cardiac vagal tone, autonomic cardiac modulation, vagus nerve, vagal tone, vagal activity, randomized controlled trial, and randomized. The specific search strategy is detailed in **Supplementary Table 1** (using PubMed as an example).

### 2.2 Inclusion Criteria

The inclusion criteria were as follows: (1) study type: RCTs with the language limited to Chinese or English; (2) study population: adults who had a mean age of ≥18 years; were free of endocrine disease, hypertension, cardiac disease, neurological disease, psychiatric disease, lung disease, liver disease, kidney disease, Parkinson’s disease, and cancer; had undergone bariatric surgery; were pregnant; had no history of smoking; (3) interventions: AT, RT, combined training (CBT), or HIIT for the intervention group and non-pharmacological intervention or maintenance of the daily lifestyle for the control group; (4) intervention period: ≥4 weeks or 8 times; (5) outcome indicators: short-range HRV analysis in the quiet state, without restriction on measurement posture (supine/sitting/standing), including the following time domain indicators: standard deviation of normal–normal intervals (SDNN), and root mean square of successive RR interval differences (RMSSD), alongside the following frequency domain indicators: high-frequency (HF) power, low-frequency (LF) power, and LF/HF power ratio (including absolute and log-transformed values of the indicators).

### 2.3 Exclusion Criteria

The exclusion criteria were as follows: (1) studies in which participants were either moderately or highly active at baseline; (2) randomized crossover studies, single-case studies, literature reviews, conference papers, and literature whose full text was unavailable; (3) repeatedly published studies.

### 2.4 Study Selection

The Endnote X20.0 software (Clarivate, Philadelphia, Pennsylvania, USA) was used to screen the included literature. First, duplicates were screened using the software’s check function, and second, two reviewers (FMY and YLS) independently read the titles and abstracts and screened them against the inclusion and exclusion criteria, and any disagreements were resolved through discussion with a third party (CW).

### 2.5 Data Extraction

The two reviewers read, evaluated, and extracted data from the articles that met the selection criteria. The main data extracted were as follows: first author, publication time, sample size, participant characteristics (age, sex, and body mass index), intervention characteristics (mode or time), HRV test plan (test method, breathing mode, and posture), research scope, and outcome index. In the order of supine, sitting, and standing postures, the three positions prevailed. When measures for various periods of interventions were included, data for longer periods of interventions were also included. When data could not be retrieved, one or another relevant author was contacted by email.

### 2.6 Risk of Bias in the Individual Studies

Two researchers independently assessed the risk of bias using the Risk of Bias 2 tool against the Cochrane Handbook version 5.3.0 for RCTs (Cochrane, London, UK). The assessment was conducted considering the following: (1) randomized sequence generation, (2) concealment of drug allocation, (3) blindness of participants, (4) personnel, (5) incompleteness of prognostic data, (6) selective reporting, and (7) influence of other factors. The risk of bias was divided into three levels: high risk, low risk, and unknown risk [34, 35].

### 2.7 Data Analysis

In this study, HRV was assessed, the effect of various exercise methods on HRV was examined, and continuous data for statistical processing were selected. Further, the data after the intervention were subtracted from the benchmark data to reflect the impact of the intervention. Considering the needs of the logarithmic transformation and functional operation, we used standardized mean differences (SMDs) instead of standard deviations. Since some differences in the initial experiment were inevitable, we selected a random approach rather than a fixed approach to obtain more scientific experimental results [36, 37].

The STATA (Version 16, StataCorp LLC, College Station Texas, College Station, TX, USA) analysis tool was used to draw an effective network evidence chart, which was required by the NMA system. In the network evidence chart, (1) each node corresponded to a kind of exercise intervention. (2) The size of the node indicated the sample size of the participants performing the intervention. (3) When there was no straight-line segment between the nodes, an indirect contrast between the nodes was noted. When a straight line existed, a straight line between the two nodes was noted. (4) The initial test sample size was represented by the thickness of the lines between the nodes. (5) The size of the node and thickness of the line segment were proportional to the number [38].

The Markov chain Monte Carlo model was used in STATA 16 to conduct the NMA and analyze the results based on the Bayesian theory. In the sorting table, it was sensible to classify the results according to the priority diagonal order. The estimated value of the paired meta-analysis was above the principal diagonal, whereas the estimated value of the NMA was below the principal diagonal [39, 40].

Finally, STATA 16.0 was used to obtain surface under the cumulative ranking curve (SUCRA) rankings and apply them to the degree of influence by the exercise interventions, with the proportion of the highest being 1 and the lowest being 0 used to measure the degree of influence by the sports activities. When the value tended to be 1, it was considered valid; otherwise, it was deemed invalid. A funnel chart on the publication deviation in the article was also generated.

## 3. Results

### 3.1 Study Identification and Selection

**Fig. 1.**
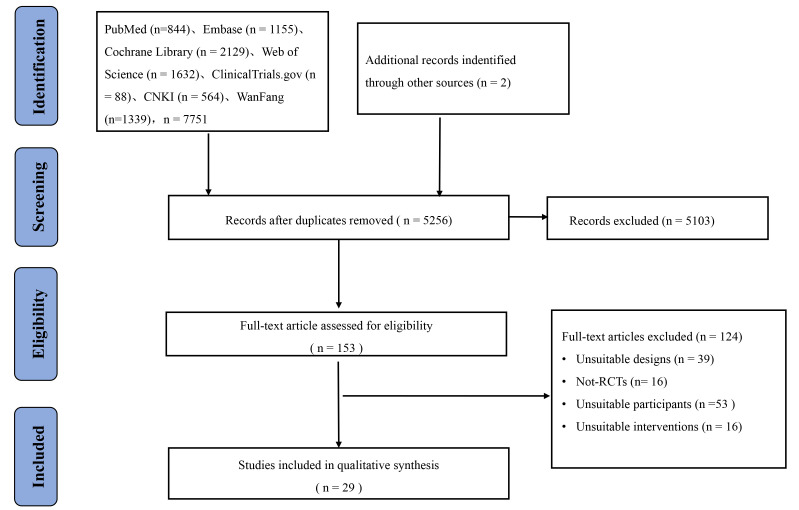
**Flowchart of literature screening**. RCTs, randomized controlled trials.

The literature search generated 7751 papers from the various databases, and an additional 2 papers were obtained manually, yielding a total of 7753 papers. After removing any duplicate papers using the literature management software (Endnote version X20), a total of 5256 original articles were obtained. After a preliminary screening of the titles and abstracts of the papers, 5103 irrelevant papers were excluded, thereby leaving 153 papers. After more in-depth reading, a further 124 papers were removed; consequently, a total of 29 original studies were included in the NMA. The article screening process is shown in Fig. 1.

### 3.2 Characteristics of the Included Studies

We included 29 RCTs, involving a total of 1317 participants [41, 42, 43, 44, 45, 46, 47, 48, 49, 50, 51, 52, 53, 54, 55, 56, 57, 58, 59, 60, 61, 62, 63, 64, 65, 66, 67, 68]. The control group did not use any interventions and only maintained daily physical activity, while the exercise group adopted four interventions: RT, HIIT, CBT, and AT. A total of 16 studies were from China, with the remaining 13 studies from other countries. Specific information on the included studies is detailed in **Supplementary Table 2**.

### 3.3 Quality Assessment of the Included Studies 

We assessed the risk of bias in each study using the Review Manager (RevMan, Version 5.3, The Cochrane Collaboration, Copenhagen, Denmark) software. Almost 35% and 65% of the studies showed a low risk and an unknown risk in random sequence generation, respectively. In allocation concealment, only 10% of the studies showed a low risk, with 90% showing a high risk. By blinding the participants and personnel data, almost 1% of the studies had a low risk, while 99% had a high risk. Conversely, almost 3% of the studies showed a low risk, while 97% showed an unknown risk when the outcome assessment was blind. For the incompleteness of the outcome data, approximately 52% showed an unclear risk, and 48% showed a high risk. In selective reporting, 100% of the studies showed a low risk. In other biases, 100% showed an unclear risk. Detailed information on the risk of bias is shown in **Supplementary Fig. 1**.

### 3.4 NMA

#### 3.4.1 LF Power

**Fig. 2.**
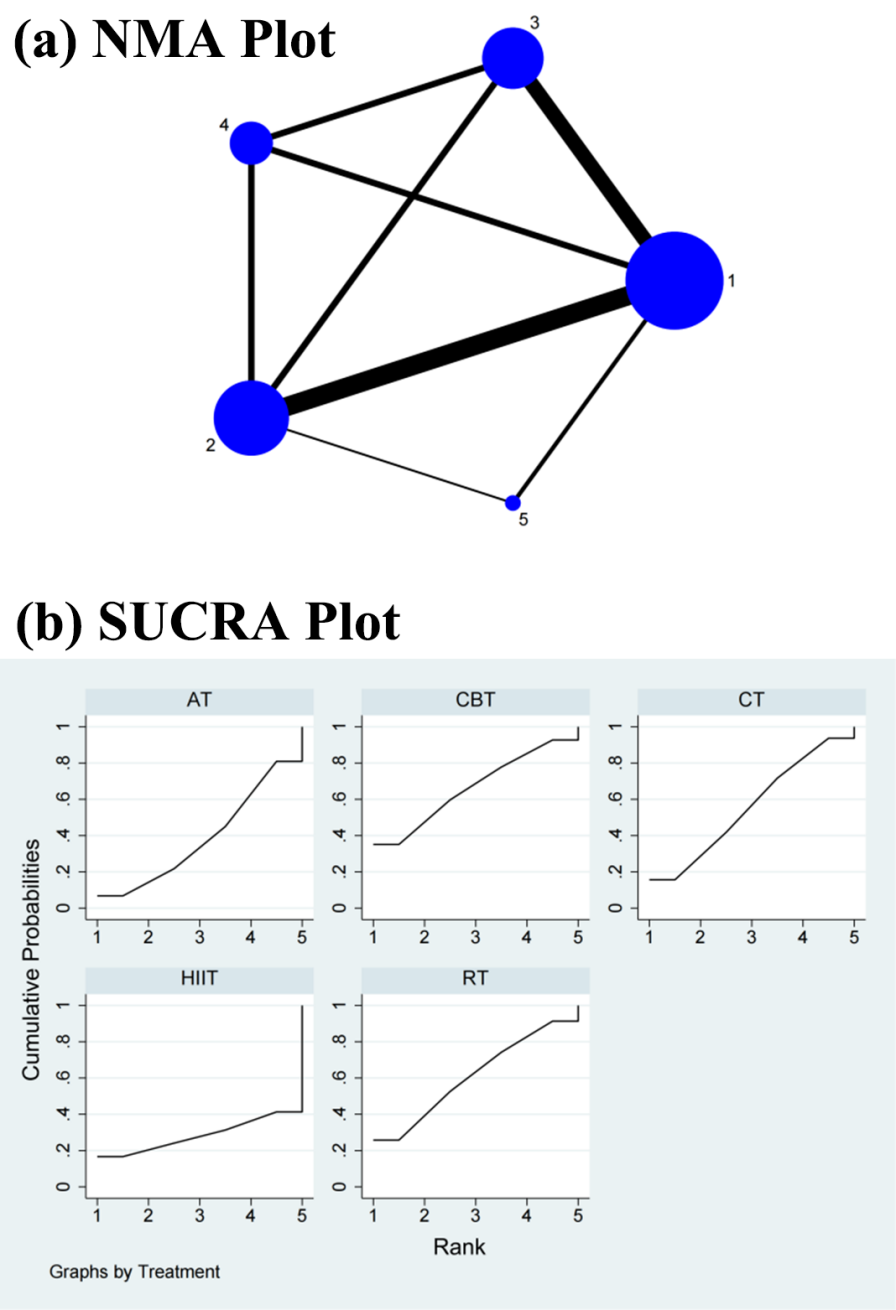
**Specific details regarding the NMA of the low-frequency power: (a) NMA plot and (b) SUCRA plot**. 1, CT; 2, AT; 3, RT; 4, CBT; 5, HIIT; NMA, network meta-analysis; CT, control treatment; AT, aerobic training; RT, resistance training; CBT, aerobic combined with resistance training; HIIT, high-intensity interval training; SUCRA, surface under the cumulative ranking curve.

**Fig. 3.**
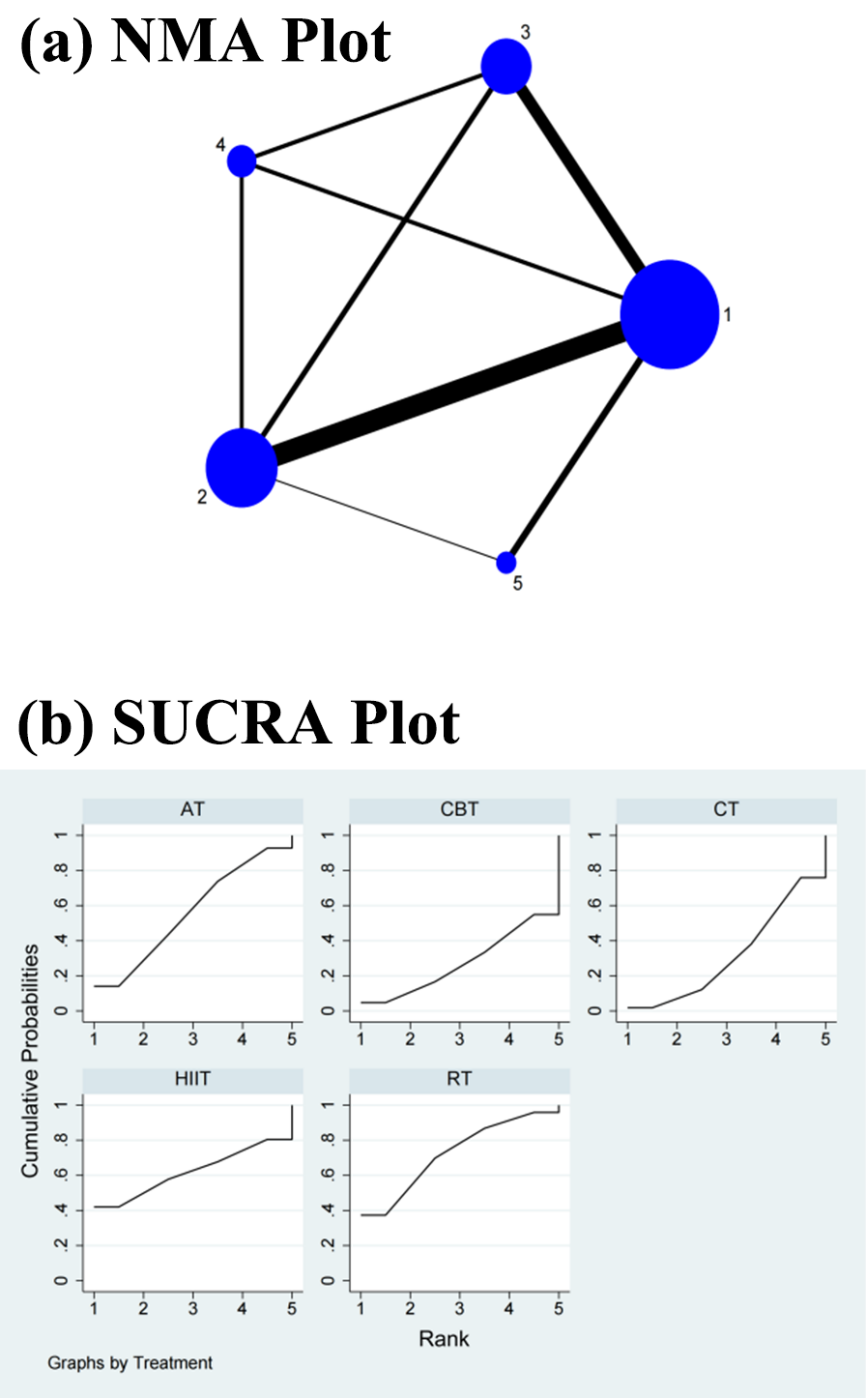
**Specific details regarding the NMA of the high-frequency power: (a) NMA plot and (b) SUCRA plot**. 1, CT; 2, AT; 3, RT; 4, CBT; 5, HIIT; NMA, network meta-analysis; CT, control treatment; AT, aerobic training; RT, resistance training; CBT, aerobic combined with resistance training; HIIT, high-intensity interval training; SUCRA, surface under the cumulative ranking curve.

**Fig. 4.**
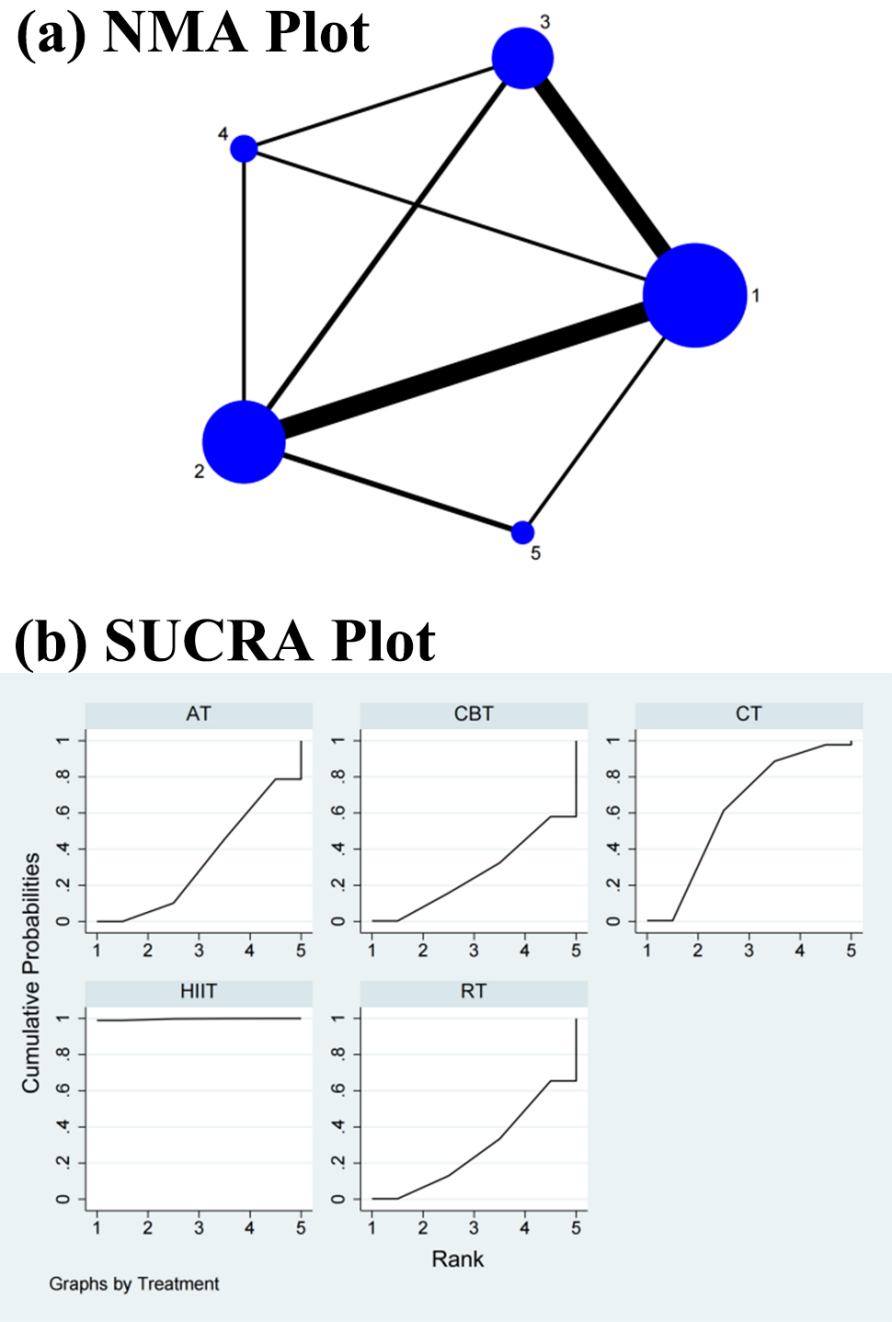
**Specific details regarding the NMA of the low-frequency/high-frequency power ratio: (a) NMA plot and (b) SUCRA plot**. 1, CT; 2, AT; 3, RT; 4, CBT; 5, HIIT; NMA, network meta-analysis; CT, control treatment; AT, aerobic training; RT, resistance training; CBT, aerobic combined with resistance training; HIIT, high-intensity interval training; SUCRA, surface under the cumulative ranking curve.

**Fig. 5.**
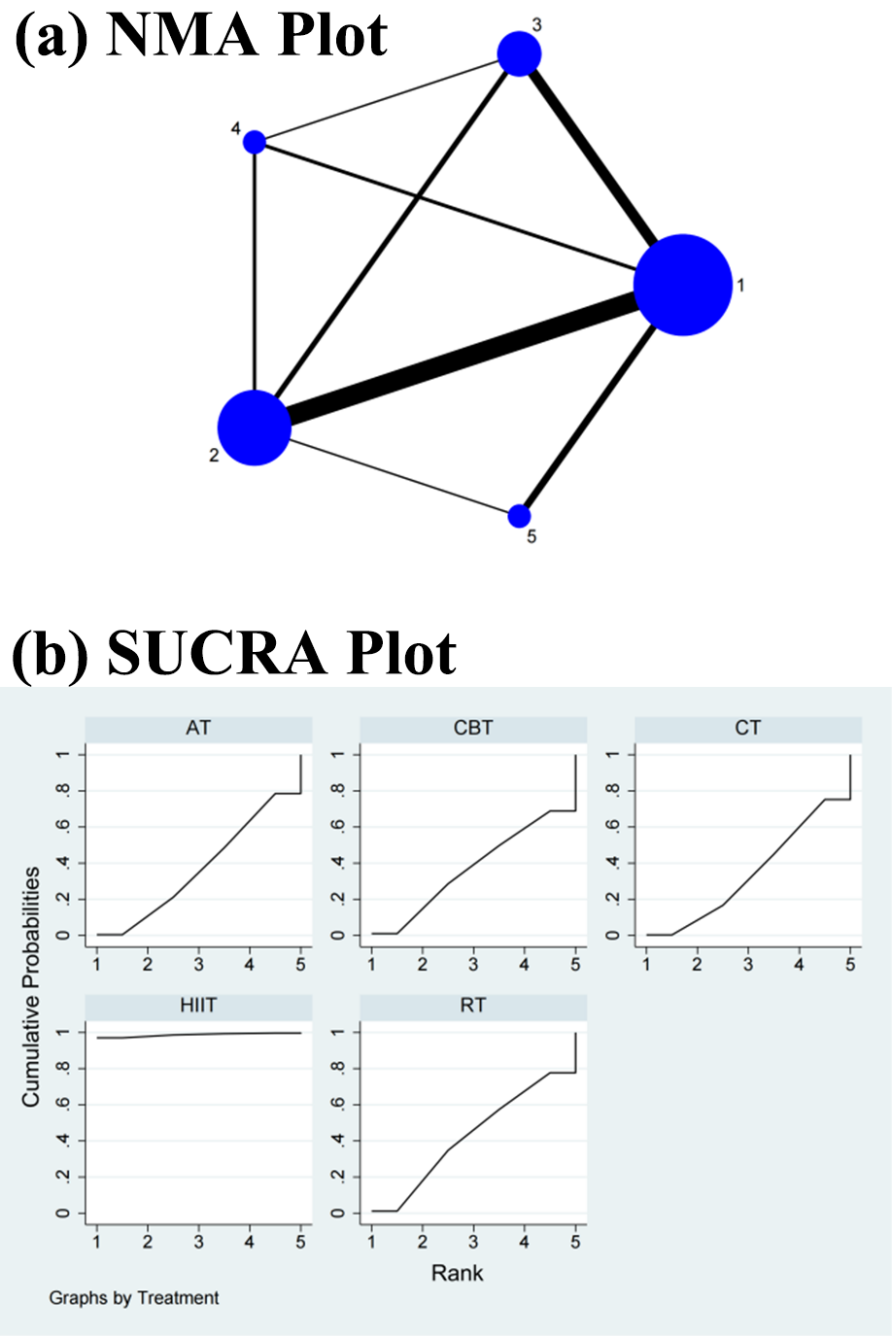
**Specific details regarding the NMA of the standard deviation of normal–normal intervals: (a) NMA plot and (b) SUCRA plot**. 1, CT; 2, AT; 3, RT; 4, CBT; 5, HIIT; NMA, network meta-analysis; CT, control treatment; AT, aerobic training; RT, resistance training; CBT, aerobic combined with resistance training; HIIT, high-intensity interval training; SUCRA, surface under the cumulative ranking curve.

**Fig. 6.**
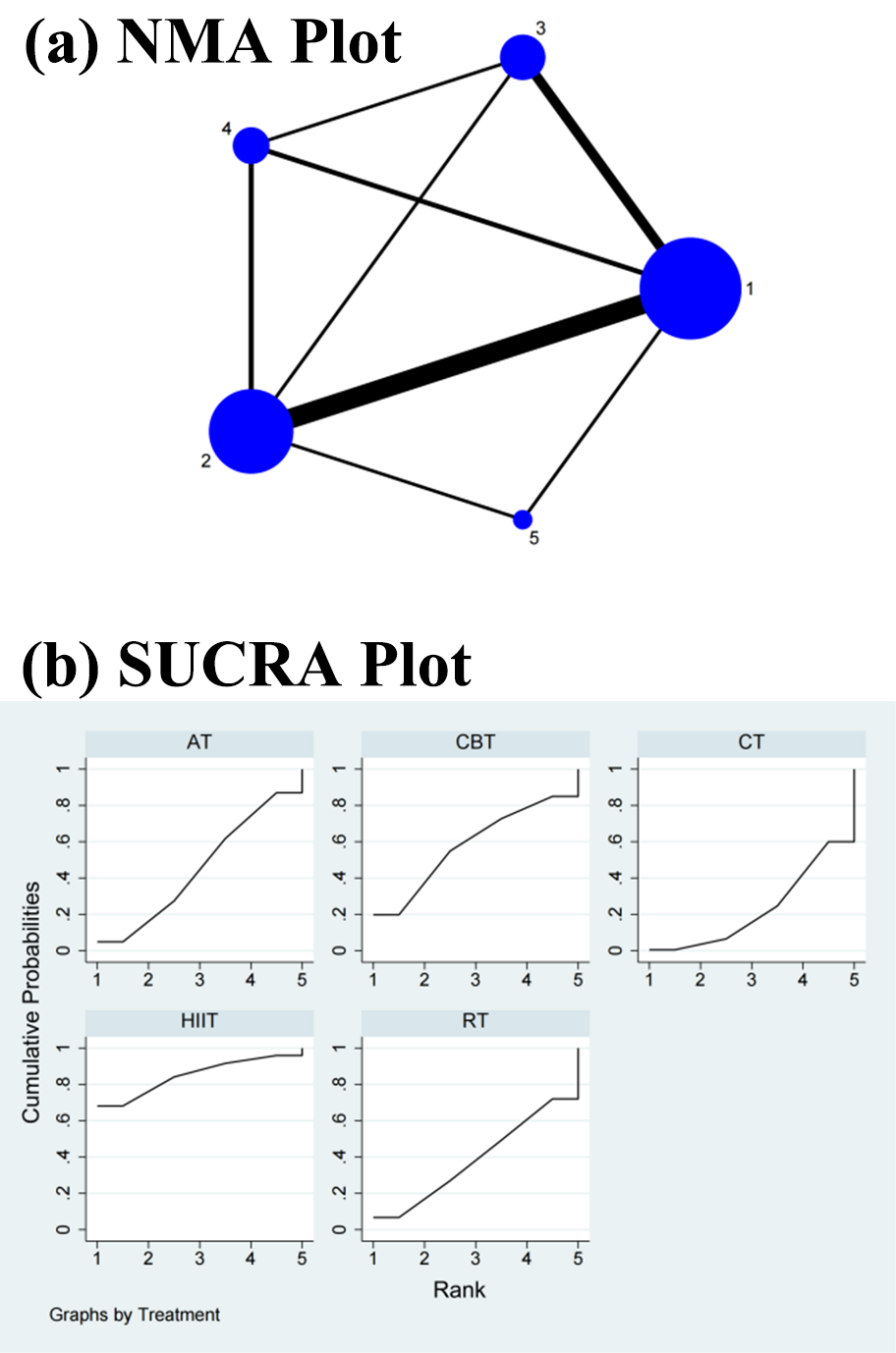
**Specific details regarding the NMA of the root mean square of successive RR interval differences: (a) NMA plot and (b) SUCRA plot**. 1, CT; 2, AT; 3, RT; 4, CBT; 5, HIIT; NMA, network meta-analysis; CT, control treatment; AT, aerobic training; RT, resistance training; CBT, aerobic combined with resistance training; HIIT, high-intensity interval training; SUCRA, surface under the cumulative ranking curve.

As shown in Figs. 2a,3a,4a,5a,6a, direct and indirect comparisons were made across the studies, and a closed loop was formed. Consistency and inconsistency assessment results showed a *p* value of >0.05, indicating that the study findings were safe and reliable. The NMA showed that in the direct comparisons the HIIT did not significantly differ from AT (SMD = –0.08, 95% CI = –0.49, 0.33), RT (SMD = –0.13, 95% CI = –0.57, 0.30), CBT (SMD = –0.15, 95% CI = –0.59, 0.29), and control treatment (CT) (SMD = –0.12, 95% CI = –0.51, 0.27). There was no significant change in the differences between the interventions in the indirect comparisons (Table 1 and **Supplementary Fig. 2**). Conversely, the SUCRA values showed that CBT ranked first in terms of efficacy in improving the LF power (SUCRA = 66.3%), followed by RT (SUCRA = 61.0%), CT (SUCRA = 55.7%), AT (SUCRA = 38.6%), and HIIT (SUCRA = 28.3%) (Fig. 2b).

**Table 1.** **League table for the low-frequency power**.

Combined training				
–0.01 (–0.23, 0.20)	Resistance training			
–0.03 (–0.24, 0.18)	–0.02 (–0.21, 0.18)	Control treatment		
–0.07 (–0.28, 0.14)	–0.05 (–0.25, 0.15)	–0.04 (–0.20, 0.13)	Aerobic training	
–0.15 (–0.59, 0.29)	–0.13 (–0.57, 0.30)	–0.12 (–0.51, 0.27)	–0.08 (–0.49, 0.33)	High-intensity interval training

#### 3.4.2 HF Power

The NMA revealed that CBT did not significantly differ from AT (SMD = 0.07, 95% CI = –0.14, 0.28), RT (SMD = 0.12, 95% CI = –0.25, 0.46), CT (SMD = 0.02, 95% CI = –0.18, 0.23), and HIIT (SMD = 0.10, 95% CI = –0.25, 0.46) in the direct comparisons. There was no significant change in the differences between the interventions in the indirect comparisons (Table 2 and **Supplementary Fig. 3**). Conversely, the SUCRA values showed that RT ranked first in terms of efficacy in improving the HF power (SUCRA = 72.5%), followed by HIIT (SUCRA = 62.0%), AT (SUCRA = 56.1%), CT (SUCRA = 32.0%), and CBT (SUCRA = 27.4%) (Fig. 3b).

**Table 2.** **League table for the high-frequency power**.

Resistance training				
0.01 (–0.33, 0.35)	High-intensity interval training			
0.05 (–0.14, 0.23)	0.03 (–0.29, 0.35)	Aerobic training		
0.09 (–0.09, 0.27)	0.08 (–0.21, 0.37)	0.05 (–0.10, 0.19)	Control treatment	
0.12 (–0.10, 0.33)	0.10 (–0.25, 0.46)	0.07 (–0.14, 0.28)	0.02 (–0.18, 0.23)	Combined training

#### 3.4.3 LF/HF Power Ratio

The NMA showed that HIIT (SMD = –0.68, 95% CI = –1.19, –0.17) differed significantly from CBT but not from RT (SMD = –0.02, 95% CI = –0.41, 0.38), CT (SMD = –0.17, 95% CI = –0.54, 0.21), and AT (SMD = –0.05, 95% CI = –0.43, 0.32) in the direct comparisons of the improvements in the LF/HF power ratio. In the indirect comparisons, there was a significant difference between HIIT and each of the other interventions, while the differences between each of the other interventions were not significant (Table 3 and **Supplementary Fig. 4**). Conversely, the SUCRA values showed that HIIT ranked first in terms of its effectiveness in improving the LF/HF power ratio (SUCRA = 99.7%), followed by CT (SUCRA = 62.1%), AT (SUCRA = 33.6%), RT (SUCRA = 28.0%), and CBT (SUCRA = 26.6%) (Fig. 4b).

**Table 3.** **League table for the low-frequency/high-frequency power ratio**.

High-intensity interval training				
**–0.52 (–0.90, –0.13)**	Control treatment			
**–0.63 (–1.00, –0.26)**	–0.11 (–0.33, 0.11)	Aerobic training		
**–0.67 (–1.14, –0.19)**	–0.15 (–0.46, 0.16)	–0.04 (–0.37, 0.29)	Resistance training	
**–0.68 (–1.19, –0.17)**	–0.17 (–0.54, 0.21)	–0.05 (–0.43, 0.32)	–0.02 (–0.41, 0.38)	Combined training

Note: The first column marked in bold indicates that the differences between high-intensity interval training and the other interventions are significant (*p* < 0.05).

#### 3.4.4 SDNN

The NMA showed that HIIT (SMD = 0.51, 95% CI = 0.09, 0.94) differed significantly from CT but not from RT (SMD = 0.02, 95% CI = –0.19, 0.24), CBT (SMD = 0, 95% CI = –0.22, 0.23), and AT (SMD = 0.01, 95% CI = –0.15, 0.16) in the direct comparisons of the improvements in the SDNN. In the indirect comparisons, there was a significant difference between HIIT and each of the other interventions, while the differences between each of the other interventions were not significant (Table 4 and **Supplementary Fig. 5**). Conversely, the SUCRA values showed that HIIT ranked first in terms of its effectiveness in improving the SDNN (SUCRA = 98.7%), followed by RT (SUCRA = 42.8%), AT (SUCRA = 37.2%), CBT (SUCRA = 37.1%), and CT (SUCRA = 34.3%) (Fig. 5b).

**Table 4.** **League table for the standard deviation of normal–normal intervals**.

High-intensity interval training				
**0.49 (0.02, 0.96)**	Resistance training			
**0.51 (0.08, 0.93)**	0.02 (–0.21, 0.25)	Aerobic training		
**0.51 (0.04, 0.98)**	0.02 (–0.24, 0.27)	0.00 (–0.23, 0.23)	Combined training	
**0.51 (0.09, 0.94)**	0.02 (–0.19, 0.24)	0.01 (–0.15, 0.16)	0.00 (–0.22, 0.23)	Control treatment

Note: The first column marked in bold indicates that the differences between high-intensity interval training and the other interventions are significant (*p* < 0.05).

#### 3.4.5 RMSSD

In the NMA, the CT did not differ significantly from HIIT (SMD = 0.26, 95% CI = –0.07, 0.58), RT (SMD = 0.04, 95% CI = –0.20, 0.28), CBT (SMD = 0.11, 95% CI = –0.18, 0.40), and AT (SMD = 0.06, 95% CI = –0.11, 0.22) in the direct comparisons of the improvement in the RMSSD. In the indirect comparisons, there was no significant change in the differences between the interventions (Table 5 and** Supplementary Fig. 6**). Conversely, the SUCRA values showed that HIIT ranked first in terms of efficacy in improving the RMSSD (SUCRA = 84.9%), followed by CBT (SUCRA = 58.1%), AT (SUCRA = 45.3%), RT (SUCRA = 38.8%), and CT (SUCRA = 22.9%) (Fig. 6b).

**Table 5.** **League table for root mean square of successive differences between adjacent normal-to-normal intervals**.

High-intensity interval training				
0.15 (–0.29, 0.58)	Combined training			
0.20 (–0.16, 0.55)	0.05 (–0.24, 0.34)	Aerobic training		
0.22 (–0.19, 0.62)	0.07 (–0.25, 0.39)	0.02 (–0.24, 0.28)	Resistance training	
0.26 (–0.07, 0.58)	0.11 (–0.18, 0.40)	0.06 (–0.11, 0.22)	0.04 (–0.20, 0.28)	Control treatment

### 3.5 Publication Bias

**Fig. 7.**
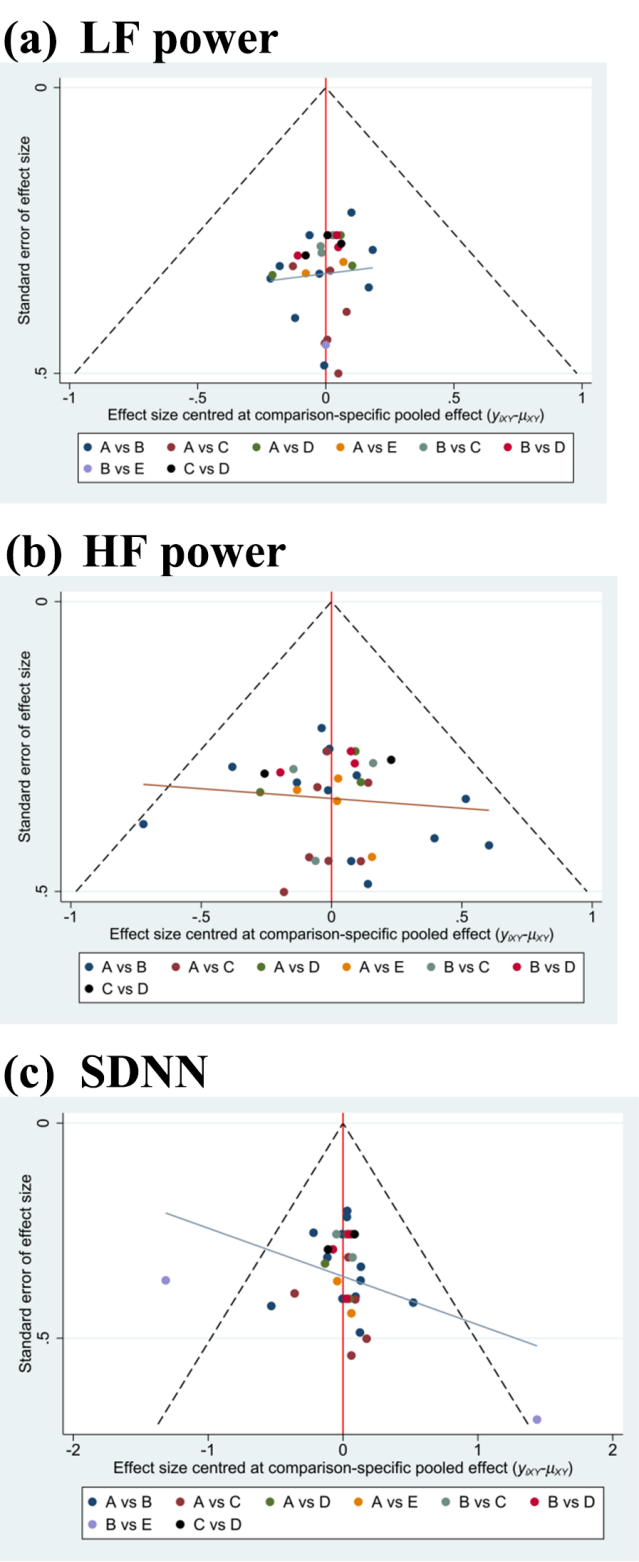
**Funnel plot to test for publication bias: (a) LF power, (b) HF power, and (c) SDNN**. A: control treatment; B: aerobic training; C: resistance training; D: combined training; E: high-intensity interval training. LF, low-frequency; HF, high-frequency; SDNN, standard deviation of normal–normal intervals.

A comparative corrected funnel plot of the LF power, HF power, and SDNN was constructed for the data evaluation using Stata 16.0. As shown in Fig. 7a–c, there was a largely symmetrical distribution on both sides of the plot, indicating that there was no effect from publication bias in the original study. The funnel plots for the RMSSD and LF/HF power ratio are shown in **Supplementary Fig. 7a,b**.

## 4. Discussion

In this study, we conducted a comparative analysis of the effects of several exercise interventions on HRV in adults. A total of 29 RCTs were found, which included four different interventions, and involved a total of 1317 participants. It was found that HIIT could increase the SDNN, RMSSD, and LF/HF power ratio in adults, while CBT and RT could increase the LF power and HF power.

Previous studies have shown that reductions in the SDNN and RMSSD are associated with an increased risk of cardiac mortality and morbidity [69, 70]. The RMSSD is an indicator of vagal tone [71]. Previous evidence has confirmed that exercise training may improve HRV in adults by decreasing sympathetic activity and increasing parasympathetic activity [72]. Heydari *et al*. [44] observed an improvement in the RMSSD in male adults after 12 weeks of HIIT (8 s sprints for 20 min, with recovery three times per week). Piras *et al*. [33] found that HIIT (20 min training followed by 1 min sprint and 2 min recovery, three times per week) increased the SDNN and RMSSD in adults. This type of training has also been shown to be more suitable for sedentary adults because of the short training time. The present NMA study also showed that HIIT was the best exercise for improving the SDNN and RMSSD. Both the direct and indirect comparisons of HIIT with the other interventions showed that HIIT was the most effective at improving the SDNN and that the difference was significant. Similarly, another meta-analysis showed that HIIT was more effective than other exercise interventions in improving HRV in adults and in increasing the SDNN and RMSSD [73]. A previous RCT also confirmed that HIIT was significantly better at improving the SDNN and RMSSD among sedentary Latin American adults than moderate-intensity continuous training (MICT) [56]. These data confirm the reliability of the results in the present study, suggesting that short periods of high-intensity exercise training may be the best-recommended form of exercise for subsequent national fitness campaigns.

Studies have shown that the LF power is associated with the activity of the stress receptor reflex system, by reflecting the complex regulation of the sympathetic and vagal nerves and, in some cases, the sympathetic nervous system tone [73, 74]. While HRV tends to decline with age, exercise increases sympathetic activity and decreases vagal activity, thereby inducing changes in the autonomic nervous system through complex metabolic and neurohumoral changes, which lead to central adaptation in the body [75]. Exercise interventions in adults can improve the decline in HRV with age and a sedentary lifestyle [76]. The results of this NMA study show that CBT is the most effective form of exercise in improving LF power. Li *et al*. [77] showed that both AT and CBT can improve the autonomic function of middle-aged and elderly people. Moreover, both of these training methods can increase vagal excitability and decrease sympathetic activity in perimenopausal female patients. However, CBT is significantly better than AT in improving the autonomic function in this population. In contrast, the results of the present NMA showed no significant differences in the direct and indirect comparisons. This finding may be partly related to the type of participants included in this study, the duration of the interventions, and the differences in age and gender.

In this study, RT was the most effective form of exercise for improving HF power, although the results of both the direct and indirect comparisons were not significant. In a study by Liu, the heart rate, HF power, LF power, and HF indices of the training group were not significantly different from those of the control group in all phases of the 12-week strength training cycle, which suggests that 12 weeks of RT does not have a significant effect on the vagal sympathetic tone [41]. In a study by Harris and Holley, in a population of prehypertensive individuals, 9 weeks of cyclic strength training did not significantly alter the resting heart rate, thereby suggesting that the training does not have any effect on parasympathetic dominance [78]. Caruso *et al*. [79] demonstrated that routine RT exacerbates atherosclerosis and that increased rates of atherosclerosis correlate with cardiac vagal pressure reflex sensitivity. Iellamo *et al*. [80] found a strong correlation between training intensity and autonomic function after 9 months of training. In their study, 1.5 months of training served as the first phase, 6 months of 75% maximal-intensity training as the second phase, and 3 months of 100% maximal-intensity training as the third phase. HRV and spontaneous stress reflex sensitivity were measured every 3 months in the second and third phases [80]. After 3 months of training at 75% intensity, there were no significant changes in the HF power, LF power, and LF/HF power ratio relative to the basal values. However, after 6 months of training at 75% intensity, there were significant increases in the RR interval, HF power, and high-frequency normalized units relative to the basal values. The increases in these indicators suggest that enhanced vagal tone occurs after 6 months of 75% intensity training. Taken together, the training duration and intensity may significantly influence HRV improvement. The inconsistencies in the intervention time and intensity in the present study resulting from the inclusion of original literature may be an important reason for the lack of any significant differences between the two groups.

The LF/HF power ratio is often used to reflect the relative activity of sympathetic and parasympathetic nerves [81]. For example, a strong sympathetic nervous system may indicate anxiety, fear, irritability, inattention, or hypervigilance. A strong parasympathetic nervous system may indicate muscle weakness, chronic neurasthenia, or depression. Some studies have shown that HIIT is more effective at improving the LF/HF power ratio than MICT [82]. This finding may be explained by the increase in vagal or pressure reflex-mediated sinus node modulation by HIIT. It has also been found that differences in the hemodynamic oscillations experienced during exercise may involve changes in the intrinsic heart rate, S–A node sensitivity [83], and/or myocardial phenotype [84]. HIIT has been shown to be an effective short-term strategy for improving cardiac autonomic function and may have important anti-arrhythmic effects [85]. The results of this NMA study also confirm that HIIT is the most effective treatment modality in improving the LF/HF power ratio and that it yields significant differences. Further, HIIT may be the best exercise modality of choice for improving the autonomic function of adults.

### Strength and Limitation

We intended to use NMA to detect HRV changes among healthy adults performing different exercise modes to provide a basis for the reasonable selection of exercise patterns. The inconsistency in the units of the HRV indicators in the included original studies might impact the final statistical analysis. To avoid such effects, we standardized the conversion of the HRV indicators used in all original studies to ensure consistency in the findings. However, there are some limitations in this study. (1) Although this study confirms the benefits of various exercise modalities on the sympathetic and vagal activities in the heart among adults, we could not classify them according to the intensity and duration of the training owing to the substantially small number of original experiments included. (2) Another limitation is the difference in the age of the participants. Most of the included participants were between the ages of 18 and 70 years. Therefore, this vast difference in age might have affected the study results. (3) Finally, a further strict limitation on the gender of the participants may result in the final NMA not being completed. Therefore, we did not strictly impose limitations on gender.

## 5. Conclusions

Our NMA and SUCRA ranking results suggest, that in healthy adults, HIIT is the most effective exercise modality for improving the SDNN, RMSSD, and LF/HF power ratio; RT and HF power; CBT and LF power. In subsequent follow-up experiments, several factors must be considered and reported, including age, sex, training intensity, frequency, and duration. In future NMA studies, subgroup analyses can also be conducted according to these factors to comprehensively explore the effects of various exercise patterns on HRV.

## Abbreviations

AT, aerobic training; RT, resistance training; CBT, combined training; HIIT, high-intensity interval training; HRV, heart rate variability; NMA, network meta-analysis; LF, low-frequency; HF, high-frequency; RCT, randomized controlled trial; MICT, moderate-intensity continuous training; SMD, standardized mean difference; CVD, cardiovascular disease.

## Supplementary Material

Supplementary material associated with this article can be found, in the online version, at https://doi.org/10.31083/j.rcm2501009.
