# Supplementary material for: Effect of Exercise Modality on Heart Rate Variability in Adults: A Systematic Review and Network Meta-Analysis
Source: Rev Cardiovasc Med. 2024 Jan 9;25(1):9. doi: 10.31083/j.rcm2501009 (PMC11262364; doi:10.31083/j.rcm2501009)
Supplement: Supplementary file 1 [file 2153-8174-25-1-009-s1.zip › 2153-8174-25-1-009-s1/Supplementary Material.docx]

**Supplementary Table 1.** Search strategy (take PubMed as an example)

| #1 | "Exercise"[MeSH Terms] |
| --- | --- |
| #2 | "exercise*"[Title/Abstract] OR "physical activit*"[Title/Abstract] OR ("activit*"[All Fields] |
| #3 | #1 AND #2 |
| #4 | ((((((((((heart rate variability[Title/Abstract]) OR (HRV[Title/Abstract])) OR (cardiac autonomic control[Title/Abstract])) OR (autonomic function[Title/Abstract])) OR (parasympathetic activity[Title/Abstract])) OR (parasympathetic nervous system[Title/Abstract])) OR (cardiac vagal tone[Title/Abstract])) OR (autonomic cardiac modulation[Title/Abstract])) OR (vagus nerve[Title/Abstract])) OR (vagal tone[Title/Abstract])) OR (vagal activity[Title/Abstract]) |
| #5 | "randomized controlled trial"[Publication Type] OR "randomized"[Title/Abstract] OR "placebo"[Title/Abstract] |
| #6 | #3 AND #4 AND #5 |

**Search strategies for other English and Chinese databases aRT as follows**

**1. Web of Science**

#1 ((((((((((((TS=(exercise*)) OR TS=(physical activit*)) OR TS=(activit*, physical)) OR TS=(exercise*, physical)) OR TS=(physical exercise*)) OR TS=(acute exercise*)) OR TS=(exercise*, acute)) OR TS=(exercise*, isometric)) OR TS=(isometric exercise*)) OR TS=(exercise*, ATrobic)) OR TS=(ATrobic exercise*)) OR TS=(exercise training*)) OR TS=(training*, exercise)

#2 ((((((((((TS=(heart rate variability)) OR TS=(HRV)) OR TS=(cardiac autonomic control)) OR TS=(autonomic function)) OR TS=(parasympathetic activity)) OR TS=(parasympathetic nervous system)) OR TS=(cardiac vagal tone)) OR TS=(autonomic cardiac modulation)) OR TS=(vagus nerve)) OR TS=(vagal tone)) OR TS=(vagal activity)

#3 ((TS=(random* controlled trial)) OR TS=(random*)) OR TS=(placebo)

#4 #1 AND #2 AND #3

1. **Embase**

#1 exercise:ti,ab,kw OR exercise*:ti,ab,kw OR 'physical activit*':ti,ab,kw OR 'activit*, physical*':ti,ab,kw OR 'exercise*, physical':ti,ab,kw OR 'physical exercise*':ti,ab,kw OR 'acute exercise*':ti,ab,kw OR 'exercise*, acute':ti,ab,kw OR 'exercise*, isometric':ti,ab,kw OR 'isometric exercise*':ti,ab,kw OR 'exercise*, ATrobic':ti,ab,kw OR 'ATrobic exercise*':ti,ab,kw OR 'exercise training*':ti,ab,kw OR 'training*, exercise':ti,ab,kw

#2 'heart rate variability':ti,ab,kw OR hrv:ti,ab,kw OR 'cardiac autonomic control':ti,ab,kw OR 'autonomic function':ti,ab,kw OR 'parasympathetic activity':ti,ab,kw OR 'parasympathetic nervous system':ti,ab,kw OR 'cardiac vagal tone':ti,ab,kw OR 'autonomic cardiac modulation':ti,ab,kw OR 'vagus nerve':ti,ab,kw OR 'vagal tone':ti,ab,kw OR 'vagal activity':ti,ab,kw

#3 random* OR placebo OR 'random* controlled trial'

#4 #1 AND #2 AND #3

**3. Cochrane Library**

#1 MeSH descriptor: [Exercise] explode all tRTes

#2 (exercise*):ti,ab,kw OR (physical activit*):ti,ab,kw OR (activit*, physical):ti,ab,kw OR (exercise*, physical):ti,ab,kw OR (physical exercise*):ti,ab,kw (Word variations have been searched)

#3 (acute exercise*):ti,ab,kw OR (exercise*, acute):ti,ab,kw OR (exercise*, isometric):ti,ab,kw OR (isometric exercise*):ti,ab,kw OR (exercise*, ATrobic):ti,ab,kw (Word variations have been searched)

#4 (ATrobic exercise*):ti,ab,kw OR (exercise training*):ti,ab,kw OR (training*, exercise):ti,ab,kw (Word variations have been searched)

#5 #1 OR #2 OR #3 OR #4

#6 (heart rate variability):ti,ab,kw OR (HRV):ti,ab,kw OR (cardiac autonomic control):ti,ab,kw OR (autonomic function):ti,ab,kw OR (parasympathetic activity):ti,ab,kw (Word variations have been searched)

#7 (parasympathetic nervous system):ti,ab,kw OR (cardiac vagal tone):ti,ab,kw OR (autonomic cardiac modulation):ti,ab,kw OR (vagus nerve):ti,ab,kw OR (vagal tone):ti,ab,kw (Word variations have been searched)

#8 (vagal activity):ti,ab,kw (Word variations have been searched)

#9 #6 OR #7 OR #8

#10 (random* controlled trial):pt OR (random*):pt OR (placebo):ti,ab,kw (Word variations have been searched) #11 #5 AND #9 AND #10

**4. China National Knowledge InfrastructuRT (CNKI)**

#1主题：运动（精确） OR 主题：活动（精确） OR 主题：锻炼（精确） OR 主题：训练（精确） OR 主题：体育（精确）

#2主题：心率变异性（精确） OR 主题：自主神经*（精确）

#3全文：随机*（精确）

#4 #1 AND #2 AND #3

**5. WanFang Data**

#1主题:(运动or 活动or 锻炼or训练 or 体育)

#2主题:(心率变异性or 自主神经or自主神经系统or迷走神经or交感神经）

#3主题:(随机or 随机分配or随机对照or随机试验or 随机对照试验)

#4 #1 AND #2 AND #3

**Supplementary Table 2**. Description of the basic characteristics of the included studies.

| Study | Location | Duration of intervention | Type | Group | n | Sex(M/F) | Age(years) | Ending indicators |
| --- | --- | --- | --- | --- | --- | --- | --- | --- |
| Badrov et al., 2013 | Canada | 8 weeks | normotensive women | EG1:IHG3 | 32 | 32/0 | 23±4 | ①②③④⑤ |
|  |  |  |  | EG1:IHG5 |  |  | 27±6 |  |
|  |  |  |  | CG:HP |  |  | /24±8 |  |
| Brown et al.,2014 | United Kingdom | 8 weeks | office workers | EG1:NWG | 94 | 74/20 | 39.3±10.3 | ④ |
|  |  |  |  | EG1:BWG |  |  | 46.3±9.4 |  |
|  |  |  |  | CG:HP |  |  | 40.2±11.0 |  |
| Gambassi et al., 2016 | Brazil | 12 weeks | healthy elderly women | EG:TG | 26 | 26/0 | 65.0±3.0 | ①②③④⑤ |
|  |  |  |  | CG:HP |  |  |  |  |
| Heydari et al., 2013 | Australia | 12 weeks | young men | EG:EG | 46 | 46/0 | 24.9±4.3 | ①②③④⑤ |
|  |  |  |  | CG:HP |  |  |  |  |
| Kanegusuku et al.,2015 | USA | 6 months | Older Adults | EG1:HIPRT | 25 | 5/7 | 64 ±4 | ③④⑤ |
|  |  |  |  | CG:CT |  | 2/11 | 63 ±4 |  |
| Karavirta et al.,2013 | Finland | 21 weeks | Middle-Aged Women | EG1:ETG | 90 | 26/0 | 52±7 | ①③④⑤ |
|  |  |  |  | EG2:STG |  | 26/0 | 52±8 |  |
|  |  |  |  | EG3:SEG |  | 21/0 | 49±6 |  |
|  |  |  |  | CG:HP |  | 17/0 | 52±8 |  |
| Karavirta et al.,2009 | Finland | 21 weeks | older men | EG1:ETG | 93 | 23/0 | 54.2 ± 11.9 | ③④ |
|  |  |  |  | EG2:STG |  | 25/0 | 56.4 ± 12.8 |  |
|  |  |  |  | EG3:SEG |  | 29/0 |  |  |
|  |  |  |  | CG:HP |  | 16/0 |  |  |
| Park et al.,2020 | Korea | 12 weeks | obesity women | EG:AEG | 36 | 18/0 |  | ①②③④⑤ |
|  |  |  |  | CG:HP |  | 18/0 |  |  |
| Prasertsri et al.,2019 | Thailand | 3 months | prehypertension | EG:ASE | 50 | 7/18 | 67.24 ± 5.29 | ①②③④⑤ |
|  |  |  |  | CG:HP |  | 4/21 | 67.32 ± 6.89 |  |
| Rodrigues et al., 2020 | Brazil | 16 weeks | overweight/obese individuals | EG1:1-HIIT | 70 | 23/47 |  | ①②③④⑤ |
|  |  |  |  | EG1:4-HIIT |  |  |  |  |
|  |  |  |  | EG:MICT |  |  |  |  |
| Shen et al.,2013 | USA | 10 weeks | Postmenopausal Women | EG:EG | 62 | 32/0 | 57.86 ± 0.64 | ①②③④⑤ |
|  |  |  |  | CG:HP |  | 30/8 | 59.10 ± 0.83 |  |
| Sloan et al.,2021 | USA | 12 weeks | healthy adult | EG:AT | 119 | 28/32 | 31.2 ± 5.7 | ②③④ |
|  |  |  |  | CG:Waitlist |  | 28/31 | 31.4 ± 6.2 |  |
| Soltani et al.,2021 | France | 12 weeks | sedentary adult men | EG:LVHIT | 51 | 17/0 | 42.2 ± 5.3 | ①②③④⑤ |
|  |  |  |  | EG:HVMIT |  | 17/0 | 42.5 ± 6.2 |  |
|  |  |  |  | CG:HP |  | 17/0 | 41.5 ± 5.6 |  |
| Verheyden et al.,2006 | Belgium | 3 weeks | healthy sedentary men | EG:TG | 29 | 14/0 | 62.4 ± 6.1 | ③④⑤ |
|  |  |  |  | CG:HP |  | 15/0 | 64.2 ± 6.5 |  |
| Wong et al.,2019 | USA | 12 weeks | Obese Postmenopausal Women | EG:RT | 20 | 0/10 | 54±3.2 | ②③④⑤ |
|  |  |  |  | CG:HP |  | 0/10 | 55±3.2 |  |
| Zlibinaite et al.,2021 | Lithuania | 2 months | Middle-Aged Women | EG:AT | 33 | 0/17 | 44.8 ± 6.5 | ②③④ |
|  |  |  |  | CG:HP |  | 0/16 | 48.8 ± 5.3 |  |
| Ramírez-Vélez et al., 2020 | Spain | 12 weeks | Inactive Adults | EG1:HIT | 20 | 3/8 | 32.1±9.6 | ①②③④⑤ |
|  |  |  |  | EG:2MCT |  | 5/4 | 31.4±6.4 |  |
| Alansare et al.,2018 | USA | 3 weeks | Inactive Adults | EG1:HIIT | 13 | 7/0 | 27.5±3.8 | ②③④⑤ |
|  |  |  |  | EG2:MICT |  | 6/0 |  |  |
| Songsorn et al.,2022 | Thailand | 4 weeks | insuffificiently active adults | EG:HIIT | 22 | 8/2 | 22.0 ± 0.8 | ①②③④⑤ |
|  |  |  |  | CG:HP |  | 7/4 | 21.7 ± 0.8 |  |
| Liu Dan et al.,2016 | China | 8 weeks | college students | EG1:SET | 30 | 10/0 | 20.41 ± 1.47 | ②③④⑤ |
|  |  |  |  | EG2:AE |  | 10/0 | 20.35 ± 0.55 |  |
|  |  |  |  | CG:HP |  | 10/0 | 20.45 ± 1.00 |  |
| Liu Dongdong et al.,2010 | China | 12 weeks | college students | EG:RT | 24 | 13/0 | NA | ①②③④⑤ |
|  |  |  |  | CG:HP |  | 11/0 |  |  |
| Liu Wei et al.,2016 | China | 24 weeks | female college students | EG1:ART | 36 | 0/12 | 20.31 ± 1.64 | ②③④⑤ |
|  |  |  |  | CG1:WE |  | 0/12 |  |  |
|  |  |  |  | CG2:HP |  | 0/12 |  |  |
| Liu zhonghao et al.,2021 | China | 2 weeks | college students | EG1:MBFRT | 32 | 8/0 | NA | ①②③④⑤ |
|  |  |  |  | EG2:LBFRT |  | 8/0 |  |  |
|  |  |  |  | EG3:RT |  | 8/0 |  |  |
|  |  |  |  | CG:HP |  | 8/0 |  |  |
| Shi hangwei et al.,2019 | China | 12 weeks | college students | EG1:CBT | 120 | 0/30 | 18.2 ± 0.41 | ①②③④⑤ |
|  |  |  |  | EG2:AE |  | 0/30 | 18.2 ± 0.41 |  |
|  |  |  |  | EG3:RT |  | 0/30 | 18.2 ± 0.41 |  |
|  |  |  |  | CG:HP |  | 0/30 | 18.2 ± 0.41 |  |
| Wang yi et al.,2016 | China | 6 months | [middle-aged and old women](file:///D:\Program%20Files%20(x86)\Youdao\Dict\9.1.6.0\resultui\html\index.html#\javascript:;) | EG1:SD | 115 | 0/15 | 60.5± 3.0 | ①②③④⑤ |
|  |  |  |  | EG2:TJQ |  | 0/24 | 59.0 ± 3.0 |  |
|  |  |  |  | EG3:RE |  | 0/15 | 60.2 ± 3.3 |  |
|  |  |  |  | EG4:SRE |  | 0/13 | 60.3 ± 3.1 |  |
|  |  |  |  | EG5:WE |  | 0/36 | 60.1 ± 2.7 |  |
|  |  |  |  | CG:HP |  | 0/12 | 60.8 ± 3.4 |  |
| Wang zhuolin et al.,2021 | China | 12 weeks | Perimenopausal Women | EG1:AE+RT | 36 | 0/12 | 51 ± 5 | ①② |
|  |  |  |  | EG2:RT |  | 0/12 | 50 ± 5 |  |
|  |  |  |  | CG:HP |  | 0/12 | 52 ± 7 |  |
| Zhao li et al.,2016 | China | 10 weeks | female college students | EG:TE | 40 | 0/14 | 21.7 ± 2.2 | ①②③④⑤ |
|  |  |  |  | CG:HP |  | 0/11 | 22.2 ± 2.0 |  |
| Zhao chunjuan et al.,2012 | China | 8 weeks | female college students | EG1:BE-4 | 34 | 0/11 | 20.73 ± 0.65 | ①②③④⑤ |
|  |  |  |  | EG2:BE-2 |  | 0/11 | 20.45 ± 0.93 |  |
|  |  |  |  | CG:HP |  | 0/12 | 20.42 ± 0.90 |  |
| Huang jianya et al.,2016 | China | 6 months | male adults | EG:HWE | 24 | 8/0 | 45.6 ± 5.3 | ①②③④⑤ |
|  |  |  |  | EG2:LWE |  | 7/0 | 45.1 ± 2.1 |  |
|  |  |  |  | CG:HP |  | 9/0 | 43.2 ± 3.3 |  |

**Note**: AEG, aerobic exercise group; AE, aerobic exercise; ART, Alternate rotation training; ASE, aerobic strength exercise; AT, aerobic training;

BE, broadcast exercise; BWG, built walking group; CG, control group; EG, exercise group; ETG, endurance training group; HIIT, high intensity interval training;

HIPRT, high intensity progressive resistance training; HP, health promotion; HVMIT, high volume moderate intensity training; HWE, high intensity

walking exercise; IH3, isometric handgrip (IHG) training; LVHIT, low volume high intensity training; LBFRT, low blood flow restriction training;

LWE, low intensity walking exercise; MBFRT, moderate blood flow restriction training; MICT, moderate intensity continuous training; NWG, normal

walking group; RE, running exercise; RT, resistance training; SET, sling exercise training; SEG, combined strength and endurance training; SD,

square dance; STG,strength training group; TE, tennis exercise; TG, training group; TJQ, taijiquan; SRE, small running exercise; WE, walking exercise;

①SDNN; ②RMSSD; ③LF; ④HF; ⑤LF/HE.

**Supplementary Fig. 1**. Risk of bias assessments for included studies


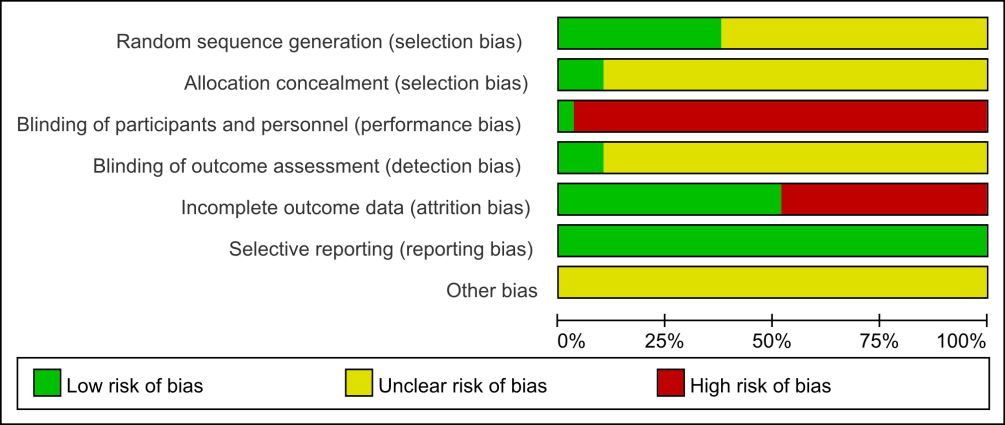


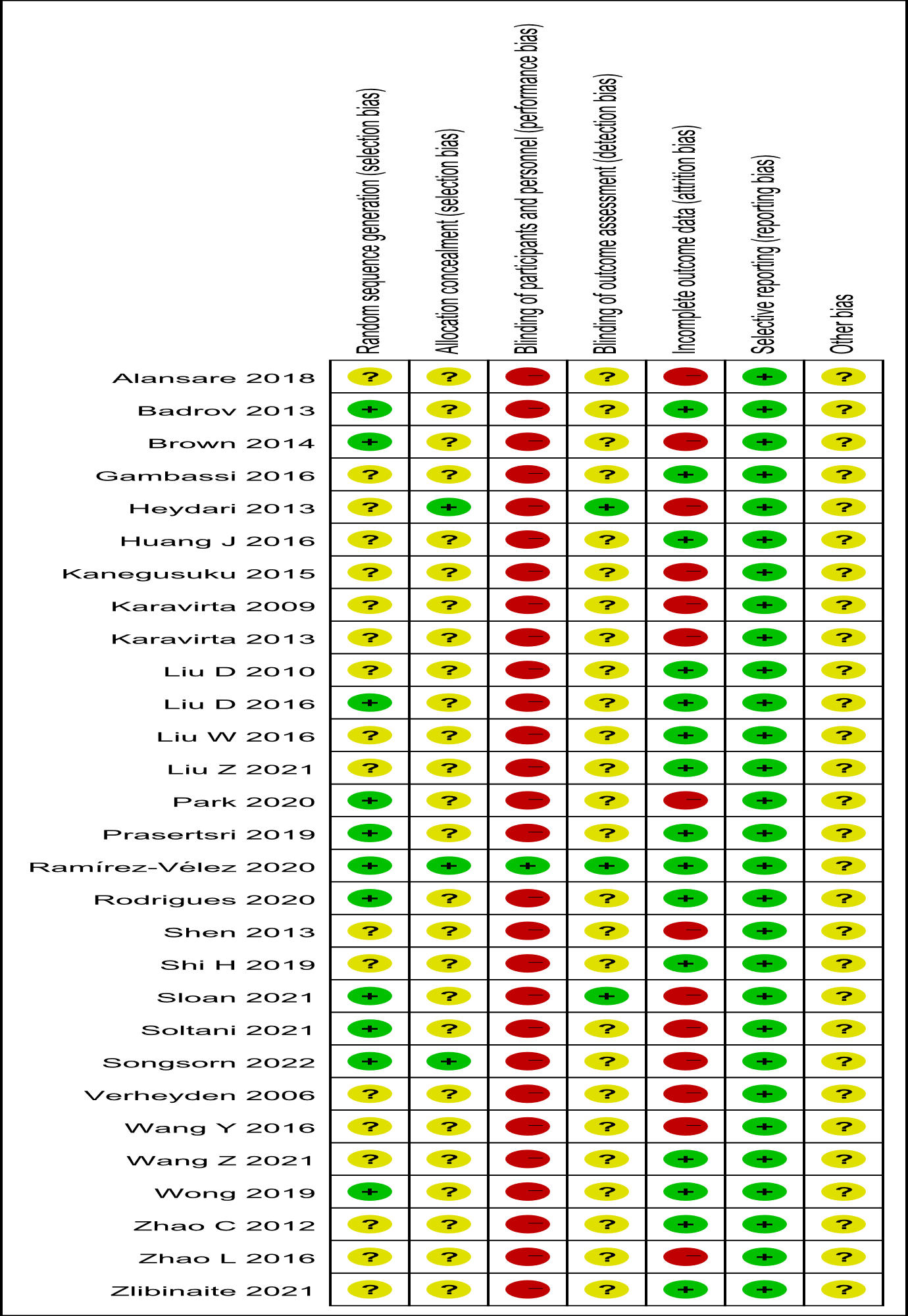


**Supplementary Figs. 2-6**


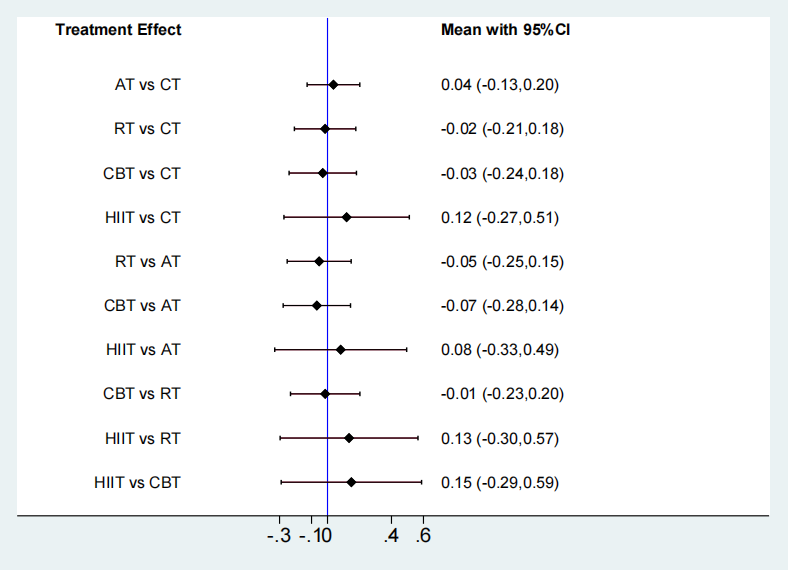


**Supplementary Fig. 2**. Direct and indirect comparisons between LF.


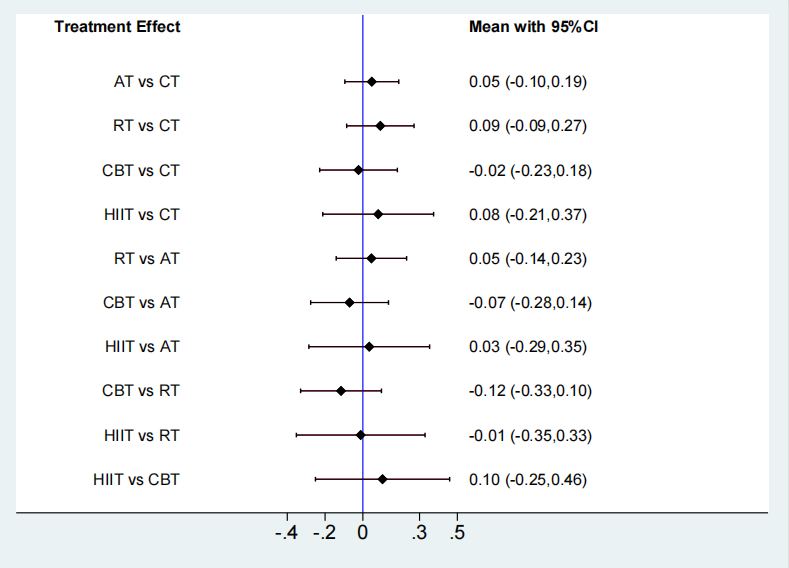


**Supplementary Fig. 3**. Direct and indirect comparisons between HF.


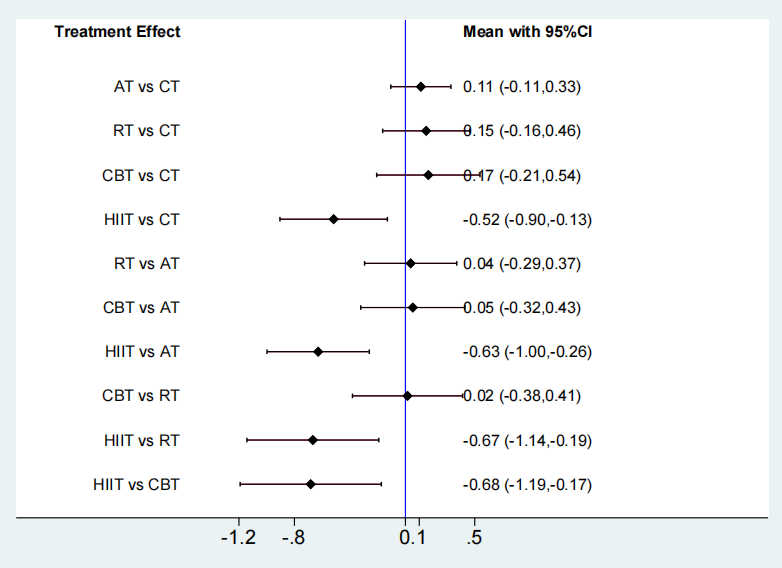


**Supplementary Fig. 4**. Direct and indirect comparisons between LF/HF.


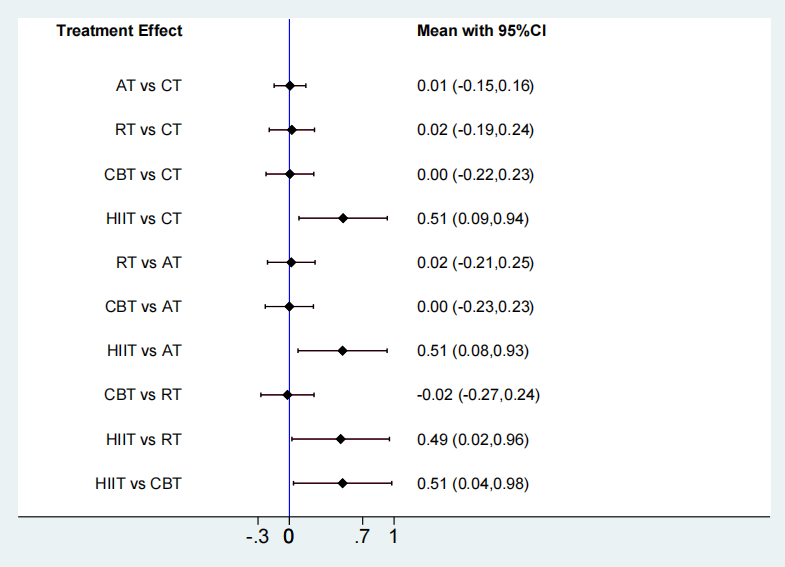


**Supplementary Fig. 5**. Direct and indirect comparisons between SDNN.


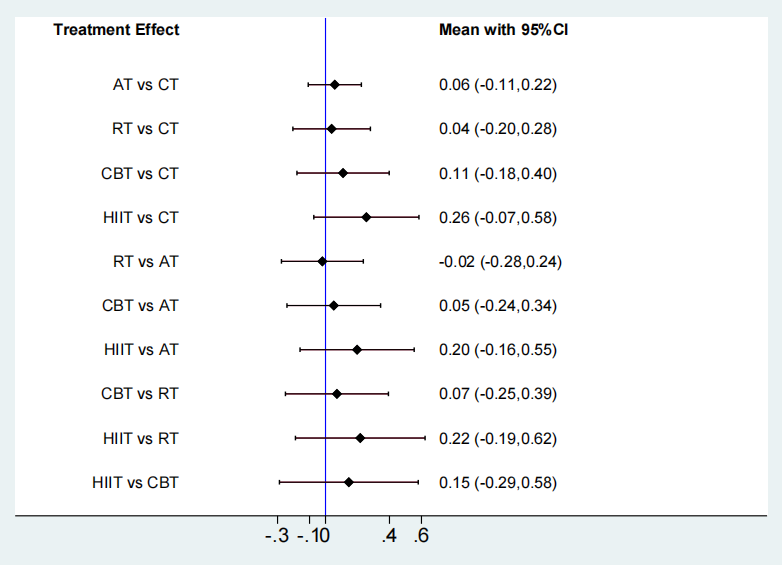


**Supplementary Fig. 6**. Direct and indirect comparisons between RMSSD.


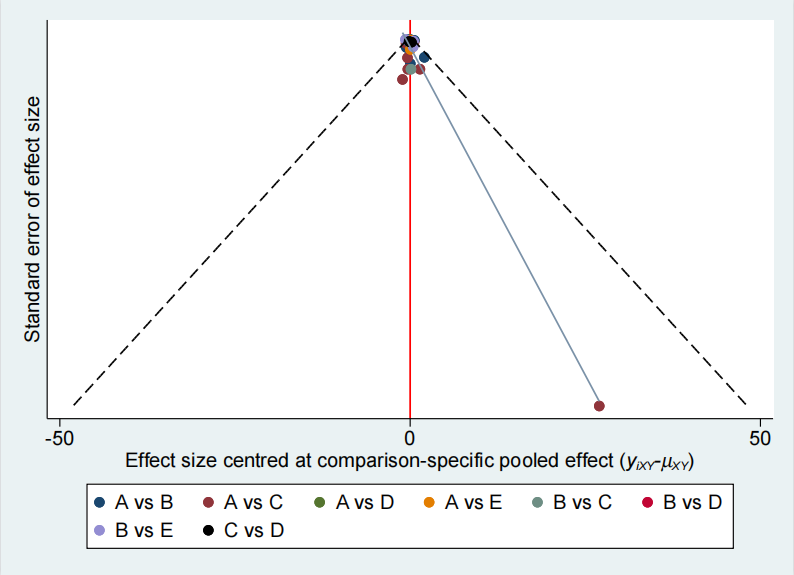


**Supplementary Fig. 7a.** Funnel plot to test for publication bias: (a) funnel plot for LF/HF. A: control treatment, B: aerobic training; C: resistance training; D: combined training; E: high intensity interval training.


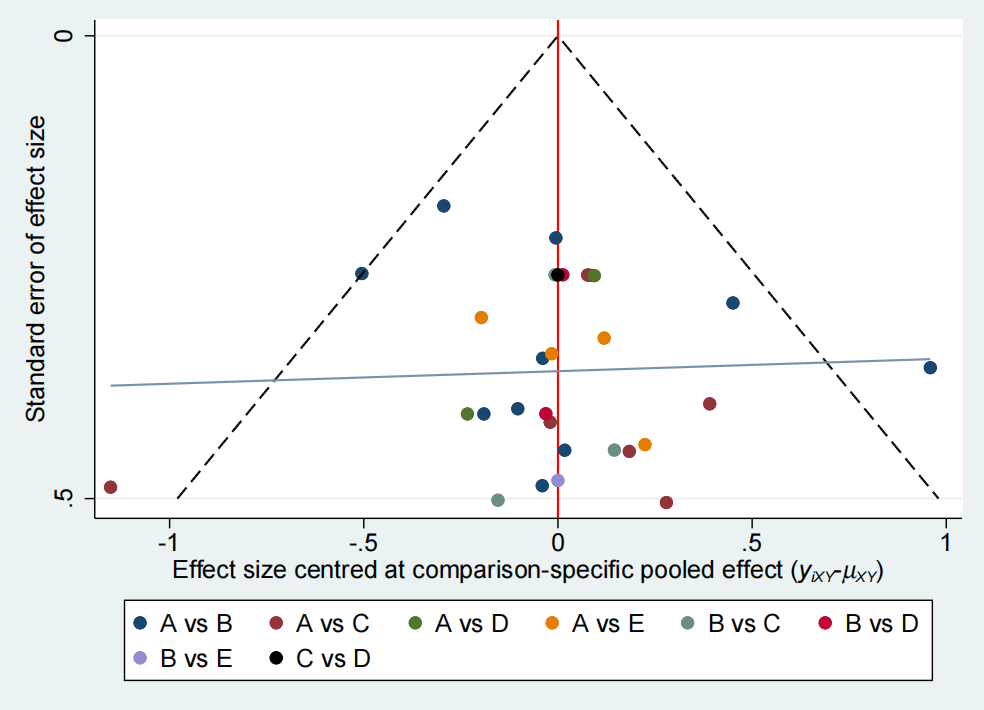


**Supplementary Fig. 7b.** Funnel plot to test for publication bias: (a) funnel plot for LF/HF. A: control treatment, B: aerobic training; C: resistance training; D: combined training; E: high intensity interval training.
